# 4031 Heart Transplant Candidates Listed at Low First-Offer Organ Acceptance Rate Centers are More Likely to Die Waiting

**DOI:** 10.1017/cts.2020.396

**Published:** 2020-07-29

**Authors:** Ashley Y Choi, Michael S. Mulvihill, Hui-Jie Lee, Congwen Zhao, Maragatha Kuchibhatla, Jacob N. Schroder, Chetan B. Patel, Christopher B. Granger, Matthew G. Hartwig

**Affiliations:** 1 Duke University; 2 Duke University Medical Center; 3 Department of Biostatistics, Duke University

## Abstract

OBJECTIVES/GOALS: We sought to examine: 1) variability in center acceptance patterns for heart allografts offered to the highest-priority candidates, 2) impact of this acceptance behavior on candidate survival, and 3) post-transplantation outcomes in candidates who accepted first rank offer vs. previously declined offer. METHODS/STUDY POPULATION: In this retrospective cohort study, the US national transplant registry was queried for all match runs of adult candidates listed for isolated heart transplantation between 2007-2017. We examined center acceptance rates for heart allografts offered to the highest-priority candidates and accounted for covariates in multivariable logistic regression. Competing risks analysis was performed to assess the relationship between center acceptance rate and waitlist mortality. Post-transplantation outcomes (patient survival and graft failure) between candidates who accepted their first-rank offers vs those who accepted previously declined offers were compared using Fine-Gray subdistribution hazards model. RESULTS/ANTICIPATED RESULTS: Among 19,703 unique organ offers, 6,302 (32%) were accepted for first-ranked candidates. After adjustment for donor, recipient, and geographic covariates, transplant centers varied markedly in acceptance rates (12%-62%) of offers made to first-ranked candidates. Lowest acceptance rate centers (<25%) associated with highest cumulative incidence of waitlist mortality. For every 10% increase in adjusted center acceptance rate, waitlist mortality risk decreased by 27% (SHR 0.73, 95% CI 0.67-0.80). No significant difference was observed in 5-year adjusted post-Tx survival and graft failure between hearts accepted at the first-rank vs lower-rank positions. DISCUSSION/SIGNIFICANCE OF IMPACT: Wide variability in heart acceptance rates exists among centers, with candidates listed at low acceptance rate centers more likely to die waiting. Similar post-Tx survival suggests previously declined allografts function as well as those accepted at first offer. Center-level decision is a modifiable behavior associated with waitlist mortality.

